# Projecting cardiovascular deaths averted due to trans fat policies in the Eurasian Economic Union

**DOI:** 10.1017/S1368980022001872

**Published:** 2023-12

**Authors:** Matthias Rieger, Holly L Rippin, Adriana Pinedo, Stephen Whiting, Clare Farrand, Kremlin Wickramasinghe, Joao J Breda

**Affiliations:** 1 International Institute of Social Studies, Erasmus University Rotterdam, The Hague, The Netherlands; 2 World Health Organization European Office for the Prevention and Control of Noncommunicable Diseases (NCD Office), Copenhagen, Denmark

**Keywords:** Industrial trans fatty acids, CVD, Trans fatty acid reduction policies, Eurasian Economic Union, WHO European Region

## Abstract

**Objective:**

To demonstrate the potential impact on population health if policies designed to reduce population trans fatty acid (TFA) intake are successfully implemented in the Eurasian Economic Union (EAEU) in line with the WHO’s guidelines to lower intake of TFA as a percentage of total energy intake to less than 1 %.

**Design:**

A projection exercise was conducted to estimate reductions in CVD-related deaths in countries of the EAEU if TFA policies are implemented in the EAEU. Plausibly causal, annual effects (in %) of Denmark’s TFA policy on the evolution of CVD mortality rates were applied to project the potential effects of recently announced TFA policies in Armenia, Belarus, Kazakhstan, Kyrgyzstan and the Russian Federation under three TFA exposure scenarios.

**Settings:**

Member States of the EAEU: Armenia, Belarus, Kazakhstan, Kyrgyzstan and the Russian Federation.

**Participants:**

Data used for the projection exercise were based on estimates from natural experimental evidence from Denmark. National CVD mortality rates used were from WHO and the Organisation for Economic Cooperation and Development datasets.

**Results:**

In all countries and in all scenarios, deaths averted were ≤ 5 deaths/100,000 in year 1 and rose in years 2 and 3. The highest projected impacts in the high-exposure scenario were seen in Kyrgyzstan (39 deaths/100 000), with the lowest occurring in Armenia (24 deaths/100 000).

**Conclusion:**

This study demonstrates the potential population health gains that can be derived from effective policies to reduce TFA in line with WHO guidance. Monitoring and surveillance systems are needed to evaluate the effectiveness of the TFA reduction policies in a national context.

Industrial trans fatty acids (iTFA) are causing approximately 540 000 deaths across the world annually^(1)^. They are artificially produced by hydrogenating vegetable or fish oils and have been widely used in the food sector as they are cheap, store well and facilitate certain aspects of baking and industrial food processing^(2)^. However, consumption of iTFA is linked to various non-communicable diseases (NCD), principally CHD^(3,4)^. Each additional 2 % in total energy obtained via iTFA is correlated with a 23 % increase in CHD incidence^(5,6)^.

In 2004, the WHO advised that consumption of trans fatty acid (TFA) as percentage of total energy intake should not surpass 1 %^(7)^. Further, WHO advocates for a total withdrawal of TFA across the world’s supply of food by 2023 and produced the REPLACE technical package, which provides several evidence-based policy options to enable countries to pursue this goal^(8)^.

In view of the evidence pointing to negative health effects of iTFA consumption and following WHO guidance, several countries have established TFA reduction policies to eliminate iTFA from the food supply. In 2004, Denmark was the first country worldwide to introduce laws against iTFA that limited TFA to 2 g per 100 g total fat^(9,10)^. Restrepo and Rieger^(9)^ used synthetic control methods to evaluate causal impacts of this ban at the population level by comparing Denmark to a weighted average of Organisation for Economic Cooperation and Development (OECD) countries with similar pre-policy CVD mortality trends. They found that in the 3 years following the TFA legislation, CVD mortality fell by 4·6 % (14·2 deaths per 100 000 people) per year. Such effects have also been echoed by recent evidence^(11)^.

A number of European countries have since implemented policies reducing TFA: Austria, Iceland, Norway, Hungary and Switzerland have introduced bans^(12)^, while others, such as the Netherlands and the UK, follow voluntary reduction strategies^(13)^. In 2019, the European Commission imposed measures limiting the iTFA content of foods intended for consumption or supply to a maximum of 2 g/100 g total fat. However, foods that do not comply may still be marketed until 2021^(14)^. In 2018, Member States of the Eurasian Economic Union (EAEU) adopted regulations restricting iTFA levels in specified oils and fat products to < 2 % of the total fat content^(15)^. Additional packaging, labelling and other measures have also been mandated^(16)^.

Implementation of the EAEU regulations will require effective monitoring and evaluation, and for this purpose, more data are needed. The WHO FEEDcities project found that the mean TFA content for widely available street food in Kazakhstan reached 144 % of the recommended maximum intake, while TFA made up half of the fat content of street food sampled in the Armenian and Kyrgyz capitals^(17)^. This supports earlier evidence from Stender *et al*.^(18)^ that products high in iTFA could be found in European cities, particularly in Eastern Europe and in smaller, ethnic shops elsewhere. In addition, Astiasarán *et al*.^(19)^ found iTFA content up to 30 % of the total fat content in some margarines.

This study builds on the available evidence examining the population-level impacts of TFA policies by conducting a projection exercise to estimate reductions in CVD-related deaths if iTFA reduction policies were enacted in Member States of the EAEU. This study specifically is based on estimates from natural experimental evidence from Denmark, which points to causal, substantial and comparable reductions in the number of CVD-related deaths following the near ban on iTFA in foods in 2003^(9,20)^. Projection exercises will be performed for Member States of the EAEU: Armenia, Belarus, Kazakhstan, Kyrgyzstan and the Russian Federation, to project the impact of effective country-level implementation of the regional policy in line with the Danish move to limit TFA to a maximum of 2 g per 100 g total fat. These projections inform the value and population health gains that can be derived from effective policies to reduce iTFA in line with WHO guidance.

## Methods

Annual effects (in %) of Denmark’s TFA policy on the evolution of CVD mortality rates were applied to project effects of similar policies in the selected countries (Armenia, Belarus, Kazakhstan, Kyrgyzstan and the Russian Federation) under three different TFA exposure scenarios.

The outcome variable was set as the CVD mortality rate from the WHO Mortality Database^(21)^, defined as the age-standardised death rates per 100 000 world standard population for both sexes^(22)^. The study on Denmark by Restrepo and Rieger^(9)^ was based on OECD (2016)^(23)^ data using an OECD-specific age standardisation, with the same ten ICD codes being used. However, the analyses in the present study used WHO data, as OECD data are currently only available for Russia. Additional results for Russia using OECD data are also presented. Absolute deaths averted will differ between the two measures available for Russia because this projection exercise assessed relative reductions following TFA legislation, and the OECD and WHO mortality rates apply different standardisations. In other words, only relative reductions are comparable.

The study period 1990–2015 was the same as in Restrepo and Rieger^(9)^ and extracted from WHO Mortality Database^(21)^. Restrepo and Rieger^(9)^ provide impact estimates for each of the 3 years following the Danish TFA policy enactment in January 2004. The projection uses an in-sample prediction within the latest possible period for all countries that provide CVD rates for at least three consecutive periods. For example, data for Russia were only available up to 2011 at the time of the analysis, resulting in a projection period of 2009–2011. Projections were compared with observed CVD rates for all countries over this period. The projection exercise was performed by applying the estimated yearly impacts of the policy (in %) in Denmark to the years 2009, 2010 and 2011 for each country.

This projection exercise assumes that legislation will be implemented as effectively in the selected study countries as in Denmark. It also assumes that TFA exposure at baseline and the resulting near elimination of TFA in these countries are comparable to Denmark in 2004 and 2009–2011. This study applies the full Danish impact estimates based on the assumption that iTFA exposure is comparable in the CVD risk groups both prior to and post-reduction. To gauge the integrity of this assumption, baseline TFA intake and trends for each of the projection countries are compared with Denmark.

In the countries studied, TFA exposure levels using popular food products high in TFA are currently only available for Kyrgyzstan (WHO, 2018)^(24)^. Consequently, projections are based on Danish data, where TFA intake is higher compared with the data available for Kyrgyzstan. In Denmark, Stender *et al*.^(25)^ provide iTFA exposure for eating at least two popular food products with a high-TFA content, such as biscuits, microwave popcorn, chicken nuggets and French fries. These data suggest a higher iTFA exposure than in the Kyrgyz case. We therefore present three scenarios (high, medium, low) to account for different potential exposure levels relative to Denmark prior to 2001. The high scenario applies the full impacts observed in Denmark, and the medium and low scenarios apply deflated impacts as detailed below:

- Scenario 1 (high) – 100 % of Danish impact estimates. 30 g/d corresponding to a high-TFA menu in Denmark prior to 2001 (Restrepo and Rieger^(9)^; taken from Stender *et al*.^(26)^)
- Scenario 2 (medium) – 59 % of Danish impacts. It is possible to consume up to 17.55 g of TFA in a typical diet in the study countries (WHO, 2018)^(24)^.
- Scenario 3 (low) – 17 % of Danish impacts. 5 g/d which is correlated with a 25 % increased risk of CHD^(27)^.

In scenario 1 (high), the entire CVD reductions (in %) in the 3 years following the TFA legislation in Denmark were applied to the study countries. These reductions were 0·9, 5·7 and 7·3 % for years 1, 2 and 3, respectively. Observed mortality rates in each of the 3 years and in each study country were multiplied by these three % reductions to calculate the number of CVD deaths averted per year; for year *t* and country *c* the projected CVD mortality rate_ct_ = the observed CVD mortality rate_ct_ × (100 %-impact_t_). In scenarios 2 (medium) and 3 (low), these % reduction impacts were deflated by multiplying them by 59 % for scenario 2 (59 % × impact_t_) and 17 % for scenario 3 (17 % × impact_t_). For example, for year 3 in scenario 2 the reduction in % is 4·3 % = 7·3 % × 59 %. It should be noted as a caveat that scenarios conflate implementation and exposure dynamics.

Table 1 presents the corresponding impact estimates that were used in the analysis, for each of the 3 years following implementation of trans fat legislation. All calculations were conducted using Excel.

**Table 1:** Annual reductions in CVD deaths following potential trans fatty acid legislation

	Treatment year		
Scenario* (% of Danish impact)	1	2	3
High (100 %)	0·9 %	5·7 %	7·3 %
Medium (Kyrgyzstan Menu, 59 %)	0·5 %	3·4 %	4·3 %
Low (17 %)	0·1 %	1·0 %	1·2 %

* Note: The ‘High’ scenario is based on the annual impact estimates (in %) from Restrepo and Rieger^(9)^. The ‘Medium’ scenario is based on a typical menu in Kyrgyzstan (WHO, 2018^(24)^) relative to a high trans fat menu in Denmark before its legislation. The ‘Low’ scenario is based on 17 % of the Danish impact estimate, which equates to 5 g/d, which is correlated with a 25 % increased risk of CHD^(27)^.

## Results

Projection results for the five selected study countries studied are summarised in Table 2. Projected impacts in the first year (2009) are below or equal to five deaths averted per 100 000 in all scenarios. Impacts rise in years 2 and 3 in all countries and across all scenarios. Deaths averted in the low scenario range from 1 to 7.

**Table 2:** CVD deaths averted: age-standardised death rates per 100 000 world standard population, WHO (2018^(21)^)

	Treatment year		
	1	2	3
	High projection*		
Armenia	3	20	24
Belarus	4	26	32
Kazakhstan	4	27	26
Kyrgyzstan	5	30	39
Russian Federation	4	29	34
	Medium projection*		
Armenia	2	12	14
Belarus	2	15	19
Kazakhstan	2	16	15
Kyrgyzstan	3	18	23
Russian Federation	3	17	20
	Low projection*		
Armenia	1	3	4
Belarus	1	4	5
Kazakhstan	1	5	4
Kyrgyzstan	1	5	7
Russian Federation	1	5	6

* Note: The projection is based on observed CVD mortality rates (rounded to whole numbers) in the years 2009, 2010 and 2011. The projection simulates annual deaths averted based on impacts presented in Table 1.

In year 3 (2011) in all scenarios projected, deaths averted per 100 000 were largest in Kyrgyzstan (scenario 1: 39), followed by Russia (scenario 1: 34). Projected deaths averted per 100 000 were lowest in Armenia and Kazakhstan (scenario 1: 24 and 26, respectively).

Table 3 gives projected impacts for Russia based on OECD data, which were also used in the Danish study. The average deaths averted were higher in Russia than in the Danish case (42 compared with 14 per 100 000) over the 3-year treatment period. Annually, in Denmark deaths averted in years 1–3 were 3; 18; 23 compared with Russia’s 8; 55; 64. This larger effect stems from the higher mortality rates evident in Russia to which the hypothetical effect of the TFA legislation is applied over the sample period and in the first year (2009) of the TFA policy (965·6 in Russia in 2009 compared with 334·3 in 2004 in Denmark, see appendix Table 1).

**Table 3:** Russian Federation (based on OECD CVD rates per 100 000 people, standardised)

	Treatment year		
	2009	2010	2011
	High projection*		
Deaths averted (rounded to whole numbers)	8	55	64
CVD mortality rate	965·60	954·70	869·10
Projected CVD mortality rate	957·39	899·85	805·38

* CVD rates and projections, age-standardised death rates per 100 000 world standard population from WHO; hypothetical legislation enactment in January 2009.

**Fig. 1:**
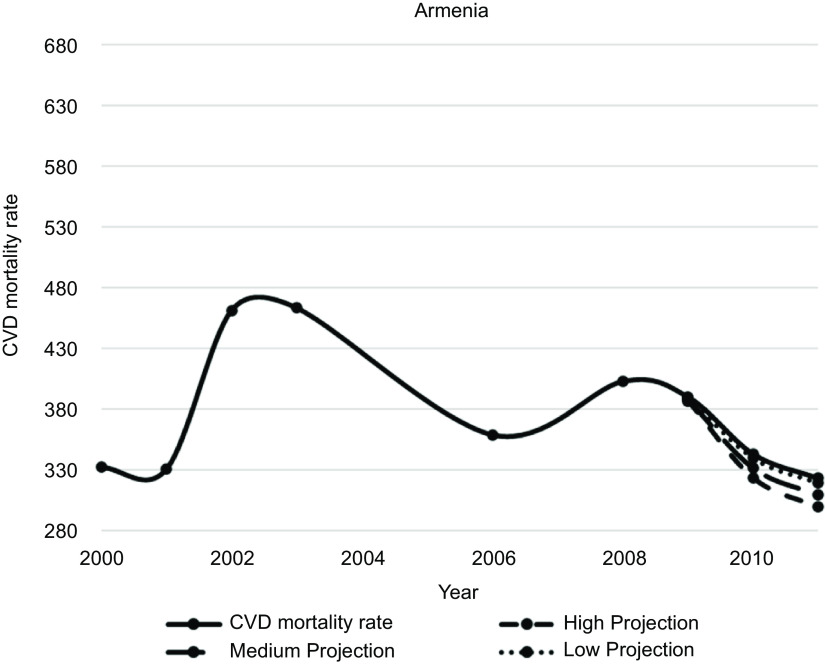
Armenia – Observed and simulated CVD mortality rate in the years 2000–2011 (age-standardised death rates per 100 000 world standard population, WHO 2018^(21)^)

**Fig. 2:**
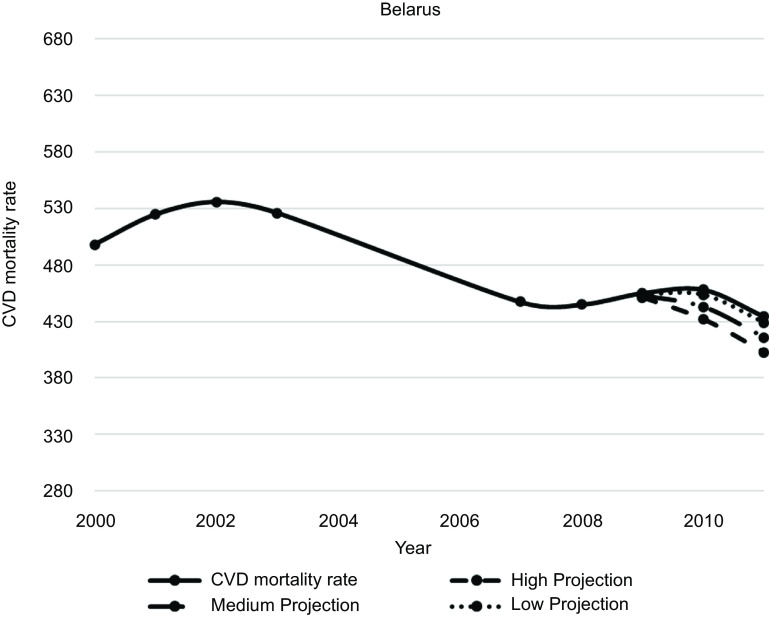
Belarus – Observed and simulated CVD mortality rate in the years 2000–2011 (age-standardised death rates per 100 000 world standard population, WHO 2018^(21)^)

**Fig. 3:**
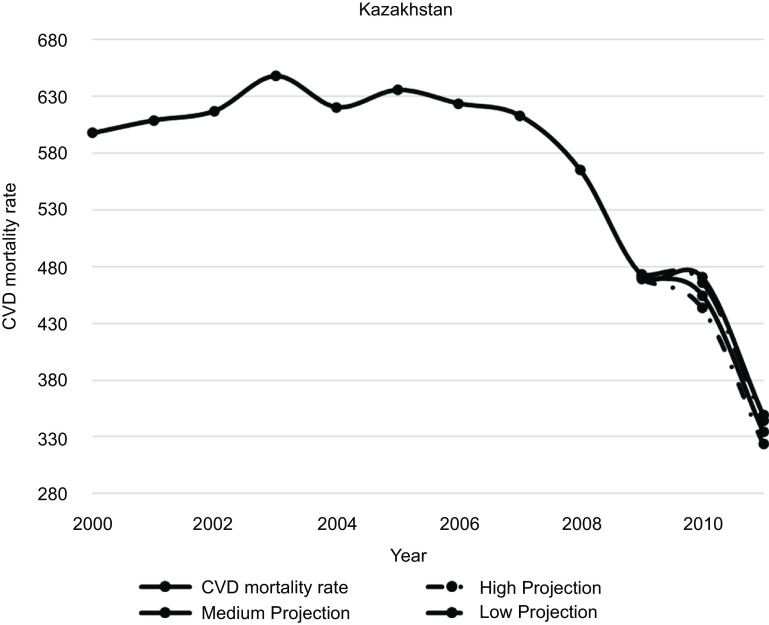
Kazakhstan – Observed and simulated CVD mortality rate in the years 2000–2011 (age-standardised death rates per 100 000 world standard population, WHO 2018^(21)^)

**Fig. 4:**
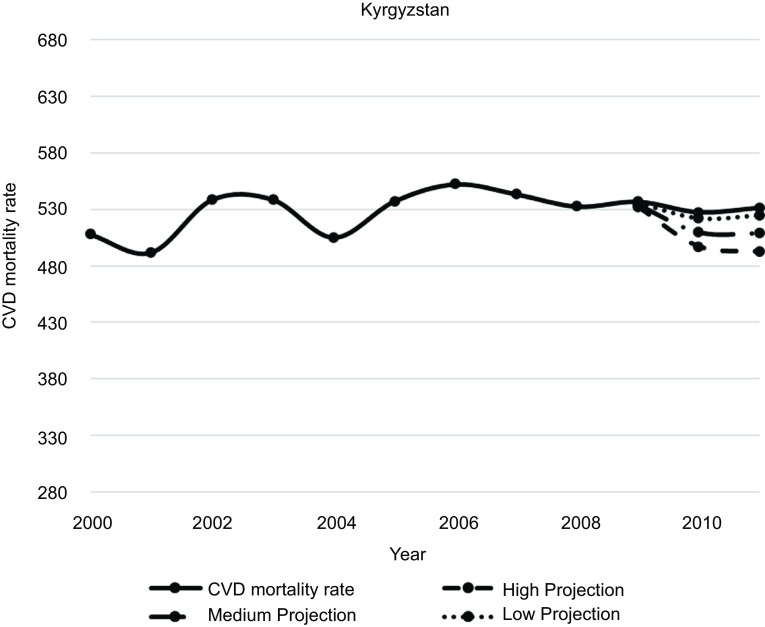
Kyrgyzstan – Observed and simulated CVD mortality rate in the years 2000–2011 (age-standardised death rates per 100 000 world standard population, WHO 2018^(21)^)

**Fig. 5:**
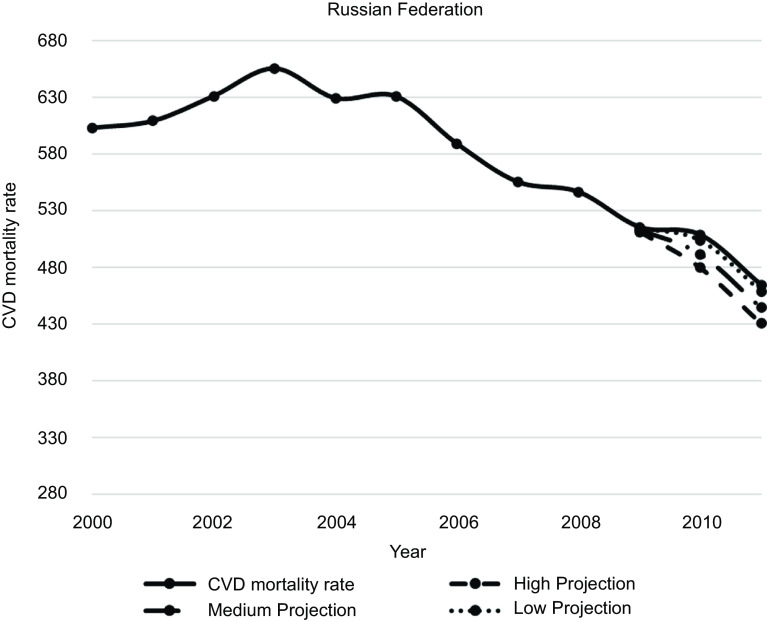
Russian Federation – Observed and simulated CVD mortality rate in the years 2000–2011 (age-standardised death rates per 100 000 world standard population, WHO 2018^(21)^)

Figures 1–5 plot CVD mortality rates against the three projection scenarios for all study countries.

## Discussion

This study adds to the existing modelling evidence that interventions for reducing iTFA have the potential to save lives. This projection study extrapolated from the implementation of a country-level policy in Denmark to estimate reductions in CVD-related deaths if equivalent TFA reduction policies had been enacted in selected Eastern European countries. Findings revealed that in all countries and in all scenarios, deaths averted rose in years 2 and 3 (2010 and 2011).

Projected impacts in the first year after enactment of TFA bans were smaller relative to subsequent years – one to five deaths averted per 100 000 – across all countries and in all scenarios due to the relatively small initial response observed in Denmark, which was in turn applied to the study countries. The Danish case suggests that treatment effects may take time to materialise. However, treatment effect dynamics may differ in the study countries compared with Denmark due to different health, food and public health/governance systems, notably, TFA exposure and implementation differences.

The highest projected impacts are seen in Kyrgyzstan across all scenarios, followed by Russia, with the lowest occurring in Armenia and Kazakhstan. Differences across countries stem from different baseline levels of CVD mortality, because annual treatment effects obtained in Denmark were used to estimate deaths averted by multiplying the observed CVD mortality with the % reduction observed in Denmark. The considerable range seen in estimated deaths averted (24–39 per 100 000 for year 3 in the high scenario in Armenia and Kyrgyzstan, respectively) demonstrates the importance of individual country context. Monitoring and surveillance systems are needed to determine the effectiveness of the TFA reduction policy in a national context, and more research into this would be beneficial in understanding these impact trends. Policies are likely to have the greatest impact in countries with high-TFA intake and the associated CVD mortality risk.

The Danish example demonstrates the life-saving potential of legislation if fully implemented. Using annual mortality rates from OECD countries for the period 1990–2012, Restrepo and Rieger^(9)^ found that 3 years after introducing the iTFA ban, CVD mortality had reduced by 4·6 % to 14·2 deaths per 100 000 compared with the synthetic counterfactual. Follow-up work has echoed these findings^(11)^. In New York, CVD mortality fell by 4·5 % following the introduction of a TFA ban^(20)^. However, evidence from elsewhere in Europe is less clear cut^(11,28)^.

Potential explanations for the success of these legislative policies include a combination of effective monitoring and enforcement mechanisms, public awareness and intersectoral collaboration. TFA bans apply to all products rather than reductions being adopted by firms engaging in a voluntary reduction process. This exposes all segments of the population to the beneficial effects, regardless of socio-economic status or nutrition and health literacy^(29)^, resulting in a population-wide reduction in CVD and related NCD^(30)^. Moreover, this policy approach is more effective and less costly for monitoring the iTFA content of foods, as this can be done at the sales level rather than measuring individual intakes^(18)^. The evidence suggests that governmental costs on TFA banning and elimination policies implementation result in substantial direct administrative saved costs over 10/15 years^(31,32)^. In Australia, it was estimated that costs related to iTFA ban policies with the main costs directed to monitoring were about USD 17M, which translated into savings 3·5 times greater over 10 years^(32,33)^. Similarly, evidence of estimated cost savings in the EU was 1·6 times greater over 15 years^(31)^. Economic gains are also seen from the reduction in NCD associated with TFA legislative bans across low-, middle- and high-income countries^(18,31)^. The closely related evaluation of trans fat bans in New York^(20)^ applied a cohort-adjusted Value of Statistical Life of USD 302,000^(35)^ and reported *‘…if fatal heart attacks cause only 1 year of life to be lost, the fatal heart attacks prevented by trans fat bans can be valued at about $465 million annually’.* Applying the same logic to our main projection (Table 1) and using WHO population estimates from 2013, Value of Statistical Life-year (VSL) estimates in year 3 and in the low projection scenario range from USD 37·35 million in Armenia to USD 2590·04 million in Russia (Table 2). While we do not have good cost estimates for our study countries, aforementioned detailed cost-benefit studies are favourable, and at least direct costs are likely smaller given evidence from Australia. At the minimum, this study’s VSL benefit range provides a useful benchmark for government’s planning and costing TFA bans.

The Danish ban was strongly influenced by evidence of health risks linked to TFA consumption. The policy’s successful introduction was aided by intersectoral collaboration between the health sector, politicians and stakeholders in the Danish food system^(36)^. Furthermore, the media facilitated public awareness of the health risks related to iTFA consumption by framing the issue as a public health concern, increasing engagement in the policy across society^(37)^.

The mechanisms contributing to the success of the Danish legislation are central to the successful implementation of an iTFA ban in the EAEU. Therefore, a roadmap encompassing similar elements and following the WHO REPLACE principles^(8)^ should be adopted to enforce the legislation successfully. To achieve effective multisectoral collaboration, the legislation will need political support. Creating awareness among policymakers may facilitate this and the enforcement of compliance with policies and regulations – a key aspect of the REPLACE package^(8)^. It may also strengthen the coordination of the policy on a regional level. Although the policy is not voluntary, ongoing collaboration with the food industry will also be required in order to identify the major sources of TFA in each country and ensure they are replaced with healthier and sustainable fats and oils. This is likely to be a slower process in Eastern Europe, leaving products containing high-TFA levels on the market for longer^(19)^. In Poland, while the availability of products containing TFA has declined, partly hydrogenated fats were used in 15 % of pastry products in 2015^(38)^.

A communication strategy should be implemented, reaching policymakers, producers, suppliers and the public^(8)^. Raising awareness is likely to enhance the reach of the policy’s success across food system actors, from large producers to street food sellers, which are popular in the countries studied, and remain a challenge in terms of TFA reduction^(17)^. Public engagement means people are more likely to adapt their consumption habits, thereby encouraging a quicker pace of change in the provision of healthier alternatives.

Finally, the legislation should be supported by robust monitoring, reporting and enforcement mechanisms. Monitoring and data collection will support robust assessments to inform policies and to reduce important risks to health^(18,28)^. This will require the strengthening of laboratory capacity and expertise, alongside the establishment of reporting channels and enforcement bodies or task forces, with penalties for non-compliance. Complimentary primary research is also needed to assess whether anticipated population health benefits are being realised and determine the impact on long-term CVD risk and NCD burden. If implemented and fully supported, such an intersectoral approach can promote national and regional environments that are healthier, affordable and culturally appropriate, where population-level NCD rates and premature deaths are reduced.

### Strengths and limitations

This is the first population-level projection exercise based on natural experiment impact estimates of the life-saving potential and population health gains that can be derived from implementation of effective trans fat policies in line with WHO guidance. A strength of this study is that it uses causal effects from a rigorous natural experiment study at the population level. This quasi-experimental design is the best option where randomised controlled trials are not possible. It is established in the literature and has been used to assess concurrent trends between Denmark and other countries and has demonstrated the effectiveness of the TFA ban at various time points^(9,11)^.

A limitation of the study is that the application of the Danish law’s estimated impacts rests on several assumptions. First, that TFA intakes were comparable at the time of the policy introduction; second, that subsequent TFA reductions following and population responses to the TFA policies were similar; finally, that the relationship between average TFA intake and CVD reduction levels is linear. If TFA intakes in the studied countries are lower than assumed, or industry response or implementation is weak, the projections based on Denmark may be overestimated.

Regarding the data, CI could not be generated because the Danish comparison study was based on a synthetic control study using aggregate data with no sampling error and randomised inference via placebo tests (i.e. false assignment of the policy to control countries and verification if the true effect ranks highly among the false effects). The present simulation could not perform such uncertainty tests, as there was no pool of control countries and placebo estimates. It also used OECD data, whereas this study used WHO data, which employ different standardisations.

Furthermore, CVD risk factors differ across Denmark and our study countries due to other factors such as alcohol consumption, salt intake and obesity. These individual risk factor differences are one caveat to keep in mind when extrapolating impacts found in one country to another country at the aggregate. Future work could extend these analyses to more Eastern European and Central Asian WHO European Member States, potentially using the PRISM tool for analysing probabilistic systems to check the projection approach^(39)^.

Additionally, further analysis of neighbouring countries following the implementation of restrictions in the EAEU in 2018^(16)^ would be useful, particularly as this could highlight potential spillover effects in both consumption and trade. With the two major trading blocs in the WHO European Region now implementing TFA bans, trading relationships could change and the price of certain high-TFA goods be affected. Further work is needed to assess the implications of this on TFA in the imported food supply and the effect on population intakes.

## Conclusion

This article conducts a projection exercise to estimate reductions in CVD-related deaths if TFA reduction policies were implemented effectively in the EAEU. It extrapolated from the implementation of a country-level policy in Denmark to estimate reductions in CVD-related deaths if equivalent TFA reduction policies had been enacted in selected Eastern European countries. The study adds to the existing modelling evidence that interventions for reducing iTFA have the potential to generate large population health gains, namely in the reduction of NCD risk. However, individual country context is key. Monitoring and surveillance systems are needed to determine the effectiveness of the TFA reduction policy in a national context. As prior stated, a limitation of the current study is that the simulations and scenarios do not distinguish between potential differences in implementation and baseline exposure to iTFA. Therefore, moving forward, more detailed, and recent data are required on exposure and potential implementation challenges to increase understanding on potential benefits in each study country.

## Appendix

**Appendix Table 1:** CVD deaths averted: age standardises death rates per 100 000 worldstandard population^(23,24)^

	CVD mortality rate (age-standardized death rates per 100 000 world standard population, WHO 2018^(21)^)					CVD mortality rate (per 100 000 people, standardized, OECD, 2016)	
Years	Armenia	Belarus	Kazakhstan	Kyrgyzstan	Russian Federation	Russian Federation	Denmark
2000	332·7	498·2	597·8	507·9	602·8	1113·2	378·6
2001	330·9	524·8	609·0	491·6	608·9	1119·1	381·8
2002	461·4	535·8	617·3	538·4	630·9	1154·2	372·3
2003	463·4	526·1	648·0	538·6	655·2	1213·7	359·9
2004			620·6	505·0	629·1	1162	334·3
2005			635·7	537·2	630·7	1164·9	313·8
2006	359·1		623·9	552·3	588·9	1099·5	297·4
2007		447·4	613·3	543·4	555·2	1043·4	278·1
2008	403·0	445·2	565·0	532·5	546·4	1023·8	257·6
2009	390·2	455·2	473·2	536·8	515·4	965·6	248·2
2010	343·3	458·3	470·6	527·5	508·7	954·7	240·6
2011	323·3	434·5	349·1	531·6	464·5	869·1	215·5
2012	322·8		282·8	533·6			212·2
2013	307·9	394·8	275·7	494·1			202·1
2014	312·7	395·4	232·2	493·6			189
2015	296·7		214·0	476·9			

**Appendix Table 2:** Value of a statistical life-year savings (in million USD)

		Treatment year	
	1	2	3
	High projection		
Armenia	28·01	186·76	224·11
Belarus	117·68	764·94	941·47
Kazakhstan	193·59	1306·76	1258·36
Kyrgyzstan	80·54	483·26	628·24
Russian Federation	1726·69	12 518·51	14 676·87
	Medium projection		
Armenia	18·68	112·05	130·73
Belarus	58·84	441·31	559·00
Kazakhstan	96·80	774·38	725·98
Kyrgyzstan	48·33	289·96	370·50
Russian Federation	1295·02	7338·44	8633·46
	Low projection		
Armenia	9·34	28·01	37·35
Belarus	29·42	117·68	147·10
Kazakhstan	48·40	241·99	193·59
Kyrgyzstan	16·11	80·54	112·76
Russian Federation	431·67	2158·36	2590·04

Value of Statistical Life-year (VSL) estimates based on Table 2, population in 2013 and the age/cohort-adjusted value of a statistical life-year of $302 000^(20,35)^.
